# Overview of Genomic Insights into Chicken Growth Traits Based on Genome-Wide Association Study and microRNA Regulation

**DOI:** 10.2174/1389202911314020006

**Published:** 2013-04

**Authors:** Zhenqiang Xu, Qinghua Nie, Xiquan Zhang

**Affiliations:** Guangdong Provincial Key Lab of Agro-Animal Genomics and Molecular Breeding, and Key Lab of Chicken Genetics, Breeding and Reproduction, Ministry of Agriculture, South China Agricultural University, Guangzhou 510642, Guang-dong, China

**Keywords:** Chicken, Genome-wide association study (GWAS), Growth traits, microRNA (miRNA) regulation, Quantitative trait loci (QTL), Single nucleotide polymorphisms (SNPs).

## Abstract

Over the two past decades, a significant number of studies have observed animal growth traits to examine animal genetic mechanisms due to their ease of measurement and high heritability. Chicken which has a significant impact on fundamental biology is a major source of protein worldwide, making it an ideal model for examining animal growth trait development. The genetic mechanisms of chicken growth traits have been studied using quantitative trait loci mapping through genome-scan and candidate gene approaches, genome-wide association studies (GWAS), comparative genomic strategies, microRNA (miRNA) regulation of growth development analysis, and epigenomic analysis. This review focuses on chicken GWAS and miRNA regulation of growth traits. Several recently published GWAS reports showed that most genome-wide significant single nucleotide polymorphisms are located on chromosomes 1 and 4 in chickens. Chicken growth, particularly skeletal muscle growth and development, is greatly regulated by miRNA. Using dwarf and normal chickens, *let-7b *was found to be involved in determining chicken dwarf phenotypes by regulating growth hormone receptor gene expression.

## 1 INTRODUCTION

The growth and development of livestock is a complicated life process and is subject to a significant amount of genetic control. Several studies have examined “hotspot” areas, including candidate gene mutation, effects of quantitative trait loci (QTL), genome-wide association studies (GWAS), microRNA (miRNA) regulation, and epigenetic inheritance. Gene expression patterns can be used to explain phenotype variations and to illustrate epigenetic mechanisms such as consensus methylation-rich promoters that correspond to down-regulation of gene expression and transgenerational stability of epigenetic variants that simultaneously appeared in mammals, birds, and plants [1-3]. Focusing on growth traits and miRNA regulation, this review discusses three main areas related to chicken growth. 

First, growth trait-correlated mutations in the genes of the somatotropic axis (Table **1**) will be discussed before focusing on GWAS and miRNA regulation of growth traits. A number of single nucleotide polymorphisms (SNPs) located within genes encoding for *growth hormone* (*GH*) and its receptor (*GHR*), *ghrelin* (*GHRL*) and its receptor (*GHSR*), *insulin-like growth factor 1 *(*IGF-1*) and its receptor (*IGF-1R*), *insulin *(*INS*),* insulin-like growth factor binding protein*2 (*IGFBP2*), *adipose triglyceride lipase* (*ATGL*), and *pituitary-specific transcription factor 1 *(*PIT1*) have been associated with chicken growth traits [4-17]. Moreover, SNPs of the *high mobility group AT-hook 2* gene (*HMGA2*), which is located on chicken (*Gallus gallus*) chromosome (GGA) 1, should be noted for their significant associations with body weight. Common insertion-deletion (indel) variations in growth-related genes have also been shown to act on growth diversity. The 1,773 bp deletion located at exon 10 and the 3′ untranslated region (UTR) of the *GHR* gene resulted in a dysfunctional precursor, leading to sex-linked dwarf chickens (SLD). One 57-bp indel in intron 2 of the *PIT1* gene was reported to be significantly associated with hatch weight and shank length at 84 d of age [8]. Another 6-bp indel of the *GHSR* gene was found to be related to crude fatty content of the leg muscle, although no evidence for its association with growth traits was identified [7]. An 8-bp indel in exon1 of the *GHRL* gene may have a negative effect on chicken growth [13]. Some SNPs are likely fixed and must transmit in a high frequency format through haplotypes such as strong linkage disequilibrium. Using haplotypes and linkage disequilibrium, important SNPs and QTL were identified. For instance, no indication of an association between either SNP A17299834G or SNP C17293932T and the *IGF-IR* gene was observed for growth traits; however, haplotypes could be constructed from these two SNPs, which were shown to be significantly associated with body weights, daily weight gains, and leg length [9]. Additionally, SNP C6540334T and SNP C6542011T of the *GHR *[10] gene and SNP A+428G of the *INS *gene [14] exhibit a similar pattern.

In addition to candidate gene approaches, identifying QTL underlying traits is a useful strategy. Continuous improvement in molecular genetics technology and DNA marker availability, identifying QTL controlling growth traits for marker-assisted selection applications has rapidly increased [18, 19]. More QTL have been identified on GGA1 and GGA4 than on other chromosomes. Among growth traits correlated with known QTL, body weight accounts for 30% (207), growth rate for 6% (40), and abdominal fat weight for 5% (35), while other traits constitute the remaining traits (http://www.animal genome.org/cgi-bin/QTL db/GG/index). For GGA4, most reported QTL are growth-related. Although several QTL have been successfully identified, most are orientated in dispersive regions of distinct chromosomes. They typically span a large range. For example, QTL affects abdominal fat weight on GGA1, which ranges from 73.6 to 215.4 cM [20] and 454 to 276 cM [21]. This makes applying these results to various commercial lines infeasible because imprecise mapping. Increasing marker density or population size under tightly restricted conditions is used for trouble-shooting. In a study by Liu *et al.*, 10 QTL were identiﬁed at the 1% chromosome-wide level for body weight from 4 to 12 weeks [22]. Shortly after, the authors refined the previously reported QTL interval by increasing nine novel microsatellites between LEI0079 and ROS0025 and enlarging family sizes from 4 to 12 and F2 individual numbers from 369 to 1011, leading to more precise QTL identification for these two traits across this region [23]. For fine mapping of this QTL, 14 additional SNPs close to ADL328 were examined and the most likely QTL for body weight was orientated between SNP G173196313A and SNP T173539900C over a 400-kb interval [24]. Finally, two SNPs (g.39692 G>A and g.77260 A>G) of the *retinoblastoma 1* (*RB1*) gene were confirmed to be related to growth. Using high-resolution QTL is effective for identifying quantitative traits near genes.

## 2 GWAS ON CHICKEN GROWTH TRAITS

The genome-wide association study, which shows a remarkable success rate, can be used to detect sequence variations influencing growth traits. GWAS on chicken growth traits showed that most significant SNPs were centered near GGA1 and GGA4 [25]. Growth traits including aggregate body weight at 0–90 d of age measured weekly, biweekly average daily gains derived from weekly body weight, and breast muscle weight, leg muscle weight, and wing weight at 90 d of age were identified in a 1.5-Mb region (173.5–175 Mb) of chicken GGA1, which was a narrow range compared to other SNPs evaluated. Of effective SNPs, five SNPs in the 1.5-Mb *karyopherin alpha 3 *(*KPNA3*)-*forkhead box O1 *(*FOXO1A*) regions showed the highest significant effects for all growth traits, while two miRNA genes putatively targeting mRNA of *IGF-1*, *FOXO1A*, and *KPNA3* genes were contained. Another GWAS study showed that most significant SNPs associated with late growth during weeks 7–12 were present in an 8.6-Mb region (71.6–80.2 Mb) of chicken GGA4, whereas only one SNP on GGA18 was significantly associated with early growth (body weight at 2 w of age). This may be due to that more genetic variance during early growth is caused by epistatic interactions rather than by single point effect [26, 27]. According to the results of artificial selection revealed in another study, a region (60–80 Mb on GGA4) may have been under intense recent selection in divergent chicken lines, where 50 generations of selection showed 9-fold differences in body weights. Similarly on GGA1, several regions respond highly to selection [28]. This implies that these two confined regions are important for understanding the molecular basis of growth traits. Although a lack of conformity between the two GWAS studies was caused by breed diversity (different materials), previously reported QTL offer powerful evidence supporting that these two regions and chicken growth are related (http://www. animalgenome.org/cgi-bin/QTLdb/GG/index). Because the described DNA variants identified through GWAS may influence growth traits, subsequent experiments should be performed [29, 30]. Of the 15 validated SNPs in the region from 173.5 to 175Mb, 12 SNPs with P<0.0033 were significant between fast-growing and slow-growing groups, indicating that these SNPs play a role in a causal mechanism or in coupling linkage phase with causal mutations [25]. 

The most promising genes harboring growth-related SNPs, including the *FOXO1A *gene, in which two highly significant SNPs at 8.9 Kb upstream and 1.9 Kb downstream were identified, may contribute to myogenic growth and differentiation. *FOXO1A *expression is notably up-regulated in energy-deprived skeletal muscle. Transgenic mice overexpressing *Foxo1a *in skeletal muscle weighed less than wild-type mice and showed reduced skeletal muscle mass [31]. The *integrator complex subunit 6* (*INTS6*) gene, containing the most significant SNP effecting aggregate body weight at 90 d of age, may act as a tumor suppressor gene by inhibiting growth of prostate cancer by altering the cell cycle profile and Wnt signaling, resulting in down-regulated expression in human multiple prostate cells [32]. The *LIM domain-binding factor 2 *(*LDB2*)gene, in which SNPs important for late growth are located, can bind to several transcription factors and drive vascular maturation [33].

SNPs in GGA1 and GGA4, which are reliably predicted using powerful statistical methods, can be used to understand chicken growth. Phenotypic differences should not only be ascribed to genetic differences or epigenetic changes as a result of sequence mutations, but also to miRNAs which affect this intricate phenomenon.

## 3 MICRORNA REGULATION OF CHICKEN GROWTH

### 3.1 MicroRNA Regulation of Biological Processes

MicroRNAs are key elements in several cell processes, including proliferation, differentiation, and apoptosis. They are a class of small non-coding RNAs approximately 22 nucleotides in length and are generally expressed in different tissues, regulating expression of specific genes at the post-transcriptional level by targeting mRNAs for degradation or inhibiting translation [34]. During miRNA formation, miRNA genes are initially transcribed as long primary transcripts (pre-miRNAs) by RNaseII or III. Mature miRNAs are generated after processing by *Dicer*, an RNase III* endonuclease*, and subsequently incorporate into the RNA-induced silencing complex (RISC) [35]. Mature miRNA always binds to the 3' UTR of target mRNAs, which mainly induces translational inhibition and exonucleolytic mRNA decay; targets can also be cleaved endonucleolytically as a result of highly complementary base-pairing [36]. Each miRNA has a specific structure and formation mechanism. For example, subtle sequence variations exist within the *gga-miR-1* and *gga-miR-133 *genes, but none exist for *gga-miR-206 *(http://www.mirbase.org/). *Gga-miR-1a *and *gga-miR-1c *differ in their 3′ ends and middle sequences by one nucleotide, while *gga-miR-133a *and *gga-miR-133c *differ in their 3′ end sequences by only one nucleotide. These sequence polymorphisms result in differential cleavage of the same miRNA precursor to produce a larger number of miRNA target sites. *Gga-miR-1a-1* and *miR-1a-2* share identical mature sequences but are located on different chromosomes. Many mRNAs can be targets of one miRNA, and different combinations of miRNAs may coordinate to regulate specific target genes [37]. For example, *miR*-1 and *miR-206* have four mutual targets: *histone deacetylase 4 *(*HDAC4*) [38, 39], *connexin43* (*Cx43*) [40],* paired box 7 *(*Pax7 *) [41], and* cMet *[42]. Additionally, *miR-206* can control myocyte differentiation by inhibiting fibroblast suppressors such as *follistatin-1 *(*Fstl1*), *utrophin *(*Utrn*) [43], and *a subunit of DNA polymerase alpha *(*Pola1*) [44], indicating its extensive role the complicated gene network.

Some miRNAs are expressed ubiquitously in different tissues, while some are expressed in a tissue-specific manner; *miR-1*, *miR-133*, and *miR-206* belong to the latter category. These muscle-specific miRNAs have been demonstrated to regulate skeletal development in different manners [38, 45]and were dramatically up-regulated during myoblast differentiation [39, 46]. Up-regulation of *miR-1* is primarily due to accumulation of myocyte enhancer factor-2 (MEF2) resulting from decreased expression of *HDAC4*; interestingly, this confined expression of *HDAC4* is caused by high *miR-1 *expression. Thus, when MEF2 expression increases accompanied by up-regulation of *miR-1*, *HDAC4 *activity is repressed, resulting in further expression of MEF2 and control of myocyte differentiation. 

### 3.2 Identification and Characterization of miRNAs Affecting Chicken Growth

Previous studies have been focused on identifying and characterizing a large number of miRNAs associated with embryo development [47, 48], skeletal muscle development [49], lipogenesis and cell proliferation [50], lung and trachea with avian influenza virus infection [51], and embryo fibroblasts infected with Marek's disease [52]. In the absence of differentially expressed miRNAs, gene expression would be dominated by monotony at the post-transcriptional and translational levels, and the resulting message would be unreadable and meaningless. Differential miRNAs found at different time points in the developing embryo and in discriminating skeletal muscle are of significant concern with regard to chicken growth. Generally, skeletal muscle goes through two stages of myoblast proliferation and differentiation during the embryonic phase and postnatal muscle fiber hypertrophy. During the embryonic phase, the appearance of somites represents muscle development. Progenitor cells derived from somites generate myoblasts, which differentiate into mature muscle fibers via proliferation, migration, and fusion [53]. Muscle growth after birth is accomplished primarily through resizing of muscle fibers resulting from satellite cell fusion to existing fibers, rather than changing their numbers, as the final number of muscle fibers is determined at the end of embryogenesis [54].

As described above, *miR-1* and *miR-133 *are involved in skeletal muscle growth and development. A bicistronic gene cluster encoding *miR-1 *and *miR-133a,* and another encoding *miR-133b* and *miR-206* are transcribed from non-coding regions on mouse chromosomes 2 and 1, respectively. Transcription of these miRNAs is mainly achieved by myogenic transcription factors, such as *myogenic differentiation 1 *(MyoD), serum response factor (SRF), and MEF2 by acting through cis-regulatory elements [55-57]. For instance, transcription regulation of *miR-1*/*133*/*206* can be implemented by mammalian target of rapamycin (mTOR) signaling in a *Myo*-dependent manner. With the aid of MyoD, which lies downstream of mTOR, transcription of *miR-1* is triggered and in turn degrades *HDAC4*, a follistatin suppressor, affecting myocyte fusion. Similarly, inhibiting mTOR by rapamycin elicits a sharp decrease in *miR-133* and *miR-206 *[58]. Moreover, *miR-1* and *miR-133* can regulate myogenesis via phosphatidylinositol 3-kinase/v-AKT murine thymoma viral oncogene (*Akt*) (PI3K/Akt) signaling Fig. (**1**), which at least partially participates in muscle growth and hypertrophy [59]. Through IGF-1 stimulation in mouse neonatal cardiomyocytes (CMCs) and during C2C12 skeletal muscle cell differentiation, binding of IGF-1 and IGF-1R results in activation of the PI3K/Akt pathways via PI3K-dependent phosphorylation and activation of Akt [60], stimulating hypertrophic growth and glucose uptake. In turn, active Akt phosphorylates and depresses transcription factor *forkhead box O3* (*Foxo3a*), a negative regulator of protein synthesis and muscle growth which regulates *miR-1* promoter activity; the declining *miR-1* reciprocally regulates IGF-1 and IGF-1R expression since they are targets of *miR-1*. This inverse correlation between IGF-1 protein levels and *miR-1* is also found in myocardial biopsies of acromegalic patients [61]. Moreover, inverse regulation of *miR-133* and IGF-1R was observed during C2C12 skeletal muscle cell differentiation [62]. Expression of *miR-133 *is up-regulated in response to increased expression of myogenin, a myogenic transcription factor which transactivates *miR-133 *in the presence of exogenous IGF-1. Overexpression of *miR-133 *has important implications on the IGF-1R/PI3K/Akt signaling pathway due to its ability to block IGF-1R expression and sequentially decrease regulation of Akt phosphorylation, *miR-1* overexpression gives rise to pronounced down-regulation of phospho-Akt supported by a generalized decrease in the IGF-1 signal transduction pathway. Another muscle-enriched miRNA, *miR-486*, reportedly indirectly and positively inhibits activation of PI3K/Akt signaling in rat CMCs through translation repression of two crucial negative regulators, phosphatase and tensin homolog (PTEN) and Foxo1a [63]. If activated by MyoD and myocardin-related transcription factor-A (MRTF-A), accumulation of *miR-486* will rise and activate PI3K, target PTEN, or activate the phosphorylation of Akt by targeting Foxo1a to activate pathway. Active Akt in reverse inhibits *Foxo1a *activity*. *Thus, *miR-1* and *miR-206 *play important roles in promoting differentiation. In contrast, *miR-133* overexpression inhibits myoblast differentiation, but promotes myoblast proliferation by targeting SRF, a regulator of *miR-133* transcription [39].

### 3.3 MicroRNAs and Embryonic Development

Dynamic sets of miRNAs have been demonstrated to express, which influence embryogenesis and organogenesis in chicks at 11 days of incubation [48]. By constructing and sequencing a small RNA library, a many known miRNAs (approximately 38% of the embryonic chick small RNA library), along with small regions of homologous miRNAs with other species and novel miRNAs, were identified. Identified miRNAs such as *miR-143*, *miR-214*, *miR-363*, *miR-10a*, and *miR-22* show a significant degree of conservation compared to mouse, gorilla, bovine, and human. At least 27 existing miRNA clusters were discovered in the chicken genome, and nearly all are conserved within vertebrate species, suggesting that miRNAs both have an evolutionally ancient origin and share similar roles among species [64]. Novel miRNAs represented 1% of identified miRNAs, while *miR-125b *accounted for 11% of the total library [48]. The most abundant *miR-125b* is significant. Most recently, *miR-125b *was implicated in myoblast differentiation *in vitro* and muscle regeneration *in vivo*. Expression of IGF-2 can be directly regulated by enhanced mTOR signaling at the transcriptional level [65]. A recent study complemented *miR-125b* to correlate IGF-2 and mTOR [66], i.e., expression of *miR-125b* is negatively controlled by mTOR signaling in an mTOR kinase-independent manner. When mTOR signaling is enhanced, *miR-125b *is reduced, and the inhibitory effect of *miR-125b* on IGF-2 is weakened, leading to high IGF-2 expression. IGF-2 is an autocrine factor that initiates myoblast differentiation *in vitro *[67]; its high expression is imperative for injury-induced muscle regeneration [68].

*In situ* hybridization of chicken embryos during early stages of incubation revealed that *miR-1*, which facilitates differentiation of mesodermal progenitors to the muscle lineage [69], was detected in the somatic myotome beginning at stage 14 [70], which corresponds to the onset of skeletal muscle cell differentiation. In contrast, *miR-133a* was detected in the myocardium and myotome at stage 15, coincident with rapid progress in limb development, and *miR-206* was detected in somites at stage 20, which is characterized by limb-bud enlargement [71]. Furthermore, restricted expression of *miR-206 *steadily increased over 1.5 to 5 d of incubation and was exclusive in developing somites, particularly in the developing myotome. An opposite trend in expression was observed following fibroblast growth factor (FGF) bead implantation due to FGF-mediated signaling, which negatively regulated the onset of *miR-206* expression [72]. FGF signals can appoint some sets of progenitor cells in the ventral somite to ribs and tendons by acting through the mitogen-activated protein kinase (MAPK) signal transduction cascade [73]. Temporal expression of specific miRNA is necessary for organ development; therefore, growth and development of the embryo can effectively occur. However, during embryogenesis and organogenesis, formation of the somite is a key step.

Somites are short-lived mesodermal structures for which ventral somite cells engender the sclerotome containing progenitor cells for cartilage and bone, and dorsal somite cells form the dermomyotome containing progenitor cells for skeletal muscle. This acts as a visible symbol for the segmented nature of the vertebrate body plan [74]. Individual vertebrae are derived from the somite, which is generated following sequential segmentation of a region of the presomatic mesoderm. Because few novel miRNAs were identified in somites using traditional sequencing, Solexa sequencing was used to globally explore novel miRNAs involving chicken somites [75]. Conventional sequencing of cDNA is a cumbersome process and cannot easily detect miRNAs present in relatively low abundance; deep sequencing or massively parallel signature sequencing (MPSS) can be used to overcome these limitations [76]. Rathjen *et al.* obtained 651,273 reads from dissected somite tissue from 3-, 4-, and 5-day old embryos and identified 42 new miRNA candidates [75]. Eighteen were confirmed using Northern blot analysis. Notably, the novel *miR-10a* was confirmed with a high number of reads (28,660), and *miR-10b *was the most abundant (113,106) among known miRNAs, implying that these two miRNAs are related during somite development. Effects caused by miRNAs on somite development and embryonic morphology have been reported previously, such as those caused by *miR-196 *[77]. *miR-196* acts upstream of the *homeobox* (*Hox*) gene to define the boundary of *Hox* gene expression along the anterior-posterior embryonic axis. A central function of the spatio-temporal expression of *Hox* gene in embryonic development is to pattern of the vertebrate axial skeleton. Unilaterally localized injection of antagomiR196 into the presomatic mesoderm in ova leads to vertebral transformations at the cervical-thoracic boundary, and thus high-frequency ectopia of the last cervical vertebra (c14) occurs as decreased *miR-196* expression gives expands the limit of *homeoboxB8 *(*Hoxb8*) transcripts in more anterior somites. Intriguingly, *miR-10* has been shown decreases expression of several *Hox* genes in zebrafish [78]. Whether this data agrees with the mechanism of regulation of *Hox* targets with *miR-196 *requires further investigation since little evidence for its role in embryogenesis has been found.

Additionally, Glazov *et al.* found that the total number of *let-7* family reads increased significantly during embryonic stages on days 5, 7, and 9, particularly *let-7b*, with maximum reads on day 9 [47]. After identification in *C. elegans*, *let-7* miRNAs have been shown to play vital roles in mediating cell proliferation and differentiation. As a lung tumor suppressor in humans, overexpression of *let-7 *results in inverse expression of *RAS*, a potential oncogene, and inhibits lung tumor cell growth [79]. It was reported to exert a negative effect on cell number and positive effect on the fraction of cells in the G2/M cell cycle phase following peripheral introduction of *let-7* in primary fibroblasts by targeting and down-regulating the *cell division cycle 34* (*Cdc34*) gene, indicating its crucial influence on cell cycle control [80]. Introduction of *let-7* family members in mouse embryonic stem cells (ESCs) can suppress continuous self-renewal resulting from a lack of *DiGeorge syndrome critical region gene 8* (*Dgcr8*), which enables silencing of this program. Moreover, inhibition of *let-7b* boosts de-differentiation of somatic cells into induced pluripotent stem (iPS) cells [81]. Overexpression of *let-7b *elevates mouse neural stem cell differentiation but reduces proliferation by targeting the stem cell regulator* nuclear receptor subfamily 2, group E, member 1* (*Nr2e1*) and the cell cycle regulator *cyclin D1*[82]. This information underscores its specific role in stabilizing the self-renewing program and differentiating stem cells. Two miRNAs, *miR-1623* and *miR-181*b, are differentially expressed in 14-day-old embryos of diverse growth-rate dwarf and normal chickens [83].

In summary, miRNAs, such as *miR-101*,play an indispensable role in establishing certain organs by localizing or fast shifting expression in appropriate timing with embryonic development. *miR-101* may monitor gonadal development to determine the gender of a chicken [84]. Expression increases between 5.5 d and 9.5 d of the embryonic phase in both sexes, with higher expression in males at E5.5 and E7.5, but a significant increase in females is observed at E9.5. It is likely that the high abundance of *miR-101* after gonadal differentiation in females is crucial for determining the nature of ovarian cells due to its inhibitory effect on *SRY (sex determining region Y)-box 9* (*SOX9*), a key component of testes differentiation. Additionally, upon suppression of some signaling inhibitory factors, transforming growth factor beta/ Anti-Müllerian hormone (TGF-β/AMH) signaling is reinforced to regulate follicle activation. In males, *miR-101* may be responsible for testes formation by fine tuning *SOX9*. As a sensitive indicator, spatio-temporal expression of miRNAs reflects the accurate developmental conditions of some organs.

### 3.4 MicroRNAs and Growth of Skeletal Muscle

Wang *et al.* identified 32 known miRNAs from skeletal muscle of Arbor Acres commercial chickens, of which 12 form five clusters: *miR-133a-1-miR-1a-2*, *miR-23b-miR-24*, *miR-99a-let-7c*, *miR-92-miR-19b-miR-18a-miR-17*, and *miR-30e-miR-30c-1*, suggesting that most miRNAs co-express in skeletal muscle [85]. Because of the complexity of muscle development based upon spatiotemporal networks regulating mRNA transcription and translation, miRNA expression during eight different developmental stages (from the birth to 7 w of age) was determined by using quantitative RT-PCR. Both *miR-133a *and *miR-1a* were more important during later stages of development than during early stages of development since expression of both increased from 14 to 49 d and decreased from 0 to 14 d.

Divergent muscle growth rate is the most common characteristic in commercial chicken lines, particularly in broilers and layers, normal, and SLD chickens. Broilers are raised for meat while layers are used for egg production, which is a consequence of the higher growth rate and larger muscle mass as well as bigger body size of broilers compared to layers. Both exterior selection pressure and intrinsic genetic patterns collectively elicit lager batteries of differences between these two breeds. For SLD chickens, aberrant muscle development attributed to a defective *GHR* gene show typical features of dwarf, serious weight loss, and decreased number of muscle fibers and fiber diameter. 

A recent preliminary study examined the miRNA transcriptome to identify differentially expressed miRNAs involved in skeletal muscle growth between broilers and layers [49]. A total of 33 novel chicken miRNAs were obtained, and 102 miRNAs showed significant differences between broilers and layers. To confirm the accuracy of these results, 17 of these miRNAs were randomly validated using microarray analysis as well as real-time RT-PCR. Expression patterns of 15 miRNAs identified using RT-PCR agreed with those identified using deep sequencing, *miR-101*, *miR-10a*, *miR-10b*, *miR-1677*, *let-7f*, and *miR-31* were higher in layers, while *miR-200b*, *let-7c*, *miR-16c*, *miR15b*, *miR-15c*, *miR460*, *miR-429*, *miR-2188*, and the novel *miR-N2 *were higher in broilers. Of note, *miR-206* was the most abundant miRNA in both broilers and layers (131,609 reads and 222,998 reads, respectively), but significant differences in expression were observed. Expression of *miR-133* was lower than that of *miR-206* and showed significant differences, *miR-1a *expression was high in both libraries and showed significant differences in expression as well. Based on this information, the corresponding mRNA transcriptome was investigated and 57 candidate targets for 16 miRNAs were identified. Additionally, the final integrated network was constructed to depict miRNA-protein interactions and protein-protein interactions. Although three miRNAs, *miR-1*, *miR-101*, and *miR-499*, were predicted to target the *activin A receptor type IIB* (*ACVR2B*) gene, validation was only performed for *miR-1* and *ACVR2B*. We examined miRNA expression in fast-growing White Recessive Rock chickens in contrast to slow-growing Qingyuan Partridge chickens at 7 w of age. We identified 23 differentially expressed miRNAs in the breast muscle of slow-growing chickens compared to in fast-growing chicken Fig. (**2A**). Wnt and insulin signaling pathways were two main pathways according to pathway analysis (data not published).

The sex-linked dwarfism chicken is characterized by its relatively small body size, lower feed intake, lower basal metabolism, reduced IGF-1 levels, and high concentrations of GH in the plasma compared to in normal chickens. Previous studies suggested that mutations in the *GHR* gene may result in phenotypic variation of SLD [86-88]. These mutations induced either different transcripts in terms of transcription level or chaotic GHR proteins in terms of translation level. Comparison of mRNA expression for *GH, GHR*, and *IGF-1* genes at 56 d of age in the livers of sex-linked dwarf chickens with normal chickens showed that GH expression between these chickens showed equally high transcription levels. However, expression of *GHR* in dwarf chickens was significantly higher than that in normal chickens, and very little *IGF-1* gene expression was observed in dwarf chickens, further illustrating that the GH-independent dwarfism is due to dysfunctional GHR protein in accordance with a mutant *GHR* gene [89]. Notably, GHR-deficient mice showed reductions in myofiber number, coupling with defective muscle skeletal development due to diminished myoblast fusion [90]. Based upon our previous study, miRNA regulation, which implicates GHR as well, may be important in dwarfism formation [83]. A significant difference in expression of *let-7b* in skeletal muscle was observed between SLD chickens due to the identical mutant type of *GHR* with Connecticut SLD [88] and normal chickens at 7 w of age. Comparison of differential expression profiles of miRNA and mRNA in SLD and normal chickens showed that, in addition to *let-7b*, mRNA expression of *GHR* genes increased SLD compared to in normal chicken. Because aberrant *GHR* elicits the dwarfism phenotype, it is important in the functional mechanism that how *GHR* acts on skeletal muscle in SLD in response to *let-7b*. The relationship between *let-7b* and *GHR* has been confirmed through a dual reporter assay and overexpression experiment *in vitro*. As expected, the complementary sequence of *let-7b* to normal *GHR *matched the deleted region of mutant *GHR*, indicating that *let-7b *suppresses expression of *GHR* in normal chickens but not in SLD chickens. The primary pathway in which *GHR *participates is for the janus kinase/signal transducers and activators of transcription (JAK/STAT) signaling pathway. Interestingly, mRNA expression of* suppressor of cytokine signaling 3 *(*SOCS3*) was markedly up-regulated in SLD in contrast to other genes involved in this pathway. Thus, tight control of *let-7b* may block expression of *SOCS3* in a non-mutant *GHR*-dependent manner.

*SOCS3* is a suppressor of cytokine signaling which blocks the JAK/STAT pathway relying on binding to janus kinase and to specific cytokine receptors, which affects cell proliferation, differentiation, apoptosis, and immunoregulation [91]. *SOCS3* also blocks insulin signaling by targeting insulin receptor substrate 1 (IRS1) and insulin receptor substrate 2 (IRS2), two critical signaling molecules for insulin action, and forces their ubiquitination and degradation, contributing to insulin resistance [92]. Leptin typically influences the feeding and neuroendocrine function depending on binding to and consequently activating its receptor (LEPR). For SLD, the indirect, acute up-regulation of *SOCS3* impairs the effect of leptin by inhibiting LEPR production. Thus, leptin suppression increases feeding and glucocorticoid production, but decreases energy expenditure of reproductive, growth, and metabolic rate. SLD chickens eat less than normal chickens, perhaps for because of their smaller somatotypes or due to unknown regulators compensating for the effect induced by *SOCS3*. However, the data provide insight into the regulatory mechanism.

Notably, disease in chicken populations can also give rise to a dramatic drop in skeletal muscle mass and result in runting and stunting syndrome (RSS). This is one of the most common syndromes causing serious growth obstruction, and chickens with this syndrome consume large amounts of feed, show poor growth performance, and can be easily affected by a disease. Thus far, its aetiology remains unclear. To identify the extensive role of miRNAs in balancing this complicated pathological mechanism, Solexa sequencing was performed to determine differential expression profiles of 22 miRNAs in the livers of normal and RSS chickens at 7 w of age Fig. (**2B**). Subsequently, we found that TGF-beta activated kinase 1/MAP3K7 binding protein 2 (TAB2) was target of *gga-miR-22*1 using a luciferase reporter gene assay Fig. (**2C** and **2D**). Interestingly, *miR-221 *affected skeletal muscle cell growth and disease. It was down-regulated during myogenic differentiation, with assistance from the RAS-mitogen-activated protein kinase (MAPK) pathway. Ectopic expression of *miR-221* in differentiated myoblasts extended the cell cycle and delayed myogenin expression, which had a suppressive role on cell cycle inhibitor p27 [93]. In chronic myeloid leukemia (CML) patients, *miR-221*was significantly up-regulated during the blast crisis phase and was predicted to target v-crk sarcoma virus CT10 oncogene homolog (avian)-like (CRKL) in the MAPK signaling and phosphoinositide-3-kinase, regulatory subunit 1 (PIK3R1) in epidermal growth factor receptor (EGFR) signaling associated with CML [94]. The roles of these two miRNAs and their targets underlying RSS must be further examined. In general, miRNAs are thought to be involved in regulating multiple genes regardless of consistently expressing or differentially expressing in different skeletal muscle by targeting and inhibiting their mRNAs. Thus, it is necessary to examine the function and characteristics of each miRNA.

## 4 CONCLUSION AND PROSPECTS

Various studies have been conducted to identify connections between genotype and phenotype. Chicken growth is complex and orchestrated through precise control of multiple genes. Association analysis using candidate gene and genome scan using linkage analysis are two primary means of detecting QTL related to growth, but this method exhibits some limitations. Because candidate genes are limited when using candidate gene approaches and QTL mapping is imprecise due to insufficient marker density when using linkage analysis, major genes leading to phenotype differences can not be determined. With the development of biotechnology and bioinformatic approaches, GWAS can be used to identify genes using tens of thousands of SNP markers at the whole-genome level. GGA1 and GGA4 are two critical chromosomes affecting chicken growth, particularly body weight. As integral members of gene networks, miRNAs explain the complexity of life. Many growth-related miRNAs have been discovered, including *miR-1*, *miR-133*, *miR-206*, *miR-101*, and *let-7b*, the biochemical roles of which have been demonstrated through experimental validation. Indepth knowledge of miRNAs will increase the understanding of the animals’ growth molecular mechanisms.

## Figures and Tables

**Fig. (1):**
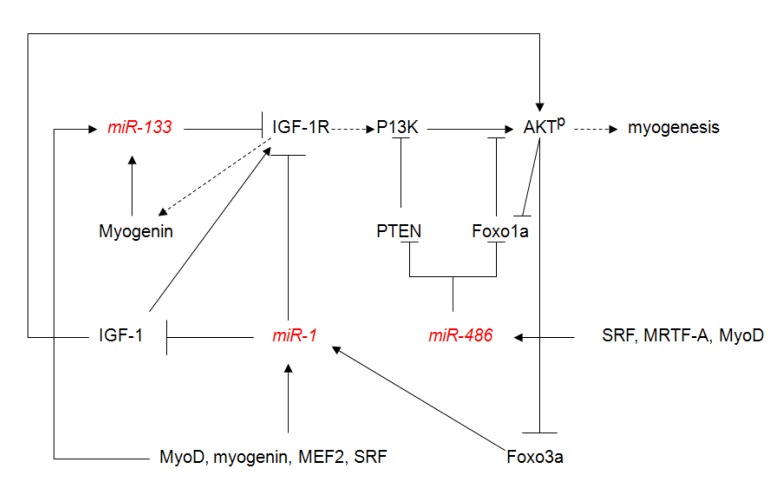
MicroRNAs regulating myogenesis via PI3K/Akt signaling. This schematic diagram is a reference for [60-63]. The dash arrows indicates the favorable pathway has not been well clarified. IGF-1: insulin-like growth factor 1; IGF-1R: insulin-like growth factor 1 receptor; AKT^P^: phospho-AKT; Foxo1a: forkhead box O1; Foxo3a: forkhead box O3; MEF2: myocyte enhancer factor-2; MRTF-A: myocardin-related transcription factor-A; SRF: serum response factor; PTEN: phosphatase and tensin homolog; PI3K: phosphoinositide-3-kinase; MyoD: myogenic differentiation 1.

**Fig. (2):**
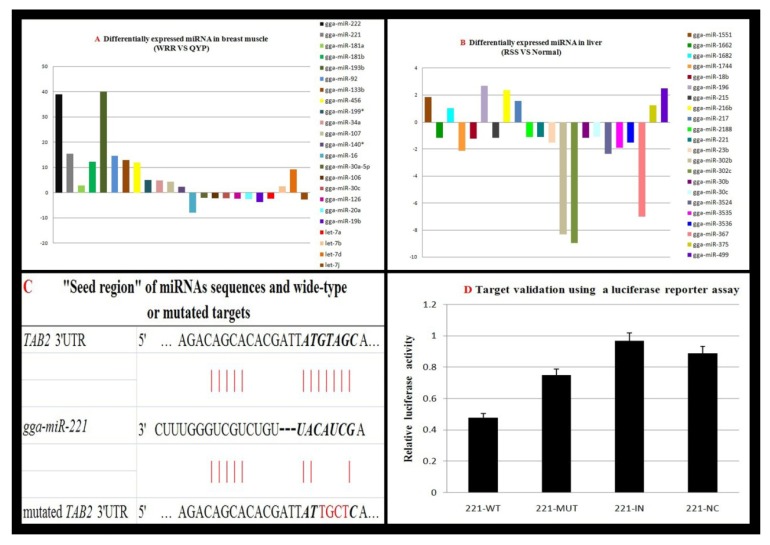
Differentially expressed miRNAs in chickens with different growth performance and target validation. **A**: Expression pattern of 23 miRNAs in breast muscles of White Recessive Rock (WRR) and Qingyuan Partridge chickens (QYP) based on microarray experiments. The bars represent the fold change in WRR/QYP. **B**: Relative abundance of 22 miRNAs in the livers of normal and RSS chickens based on Solexa sequencing data. The bars represent the fold change of RSS/normal. **C**: Schema of *gga*-*miR*-*221* binding sites in chicken wide-type and mutated TAB2 (TGF-beta activated kinase 1/MAP3K7 binding protein 2) 3'-UTR sequence. Seed binding sites and mutated bases are highlighted in red. Target prediction was performed using TargetScan 5.0 software *(http://targetscan.org/)*. **D**: To verify whether *gga*-*miR*-*221* targets *TAB2*, a luciferase reporter assay was conducted. A *gga*-*miR*-*221* overexpression plasmid was constructed using pcDNA3.1 (Promega, Fitchburg, WI, USA). Wild-type (WT) or mutated *TAB2* 3'-UTR sequence (MUT) was cloned into the pmirGLO luciferase reporter vector at downstream of *Renilla* luciferase, the internal Firefly luciferase gene was used to normalize for transfection efficiency. DF-1 cells were used for transfection, and 600 ng of the *gga*-*miR*-*221* overexpression plasmid was co-transfected with 200 ng of WT or MUT reporter plasmid (221-WT/-MUT). A parallel inhibition or negative control experiment was conducted using30 ng IN or NC vector plasmid cotransfected with 200 ng of WT reporter plasmid (221-IN/-NC). Dual-luciferase assays were conducted in triplicate in 24-well plates. Cells were lysed and assayed for luciferase activity 36h after transfection. Error bars represent the standard error of three independent experiments per group.

**Table 1.** Significant Associations of the SNPs Within Candidate Genes with their Growth Traits (P<0.01)

Gene	Chr	SNP^a^	Growth Traits^b^	Ref.

***GHRL***	7	C71T	BW: 7 wk, 16 wk;	11
			SG: 7 wk, 16 wk; SL: 16 wk	
		G1215A	BW: 16 wk	13
		8bp indel (exon)	BW: 2 wk, 3 wk, 5 wk, 6 wk, 7 wk, 13 wk; BL	

***INS***	5	T+3737C (intron)	SIL	14
		A+3971G (intron)	BW: 4 wk; HW; BA; SIL	
		C+1549T (intron)	BW: 4 wk, 12 wk; BD	

***GH***	1	G+119A (intron)	SIL	15
		G+1705A (intron)	BW: 3 wk, 4 wk, 10 wk, 12 wk; ADG: 0-4 wk;	
		G+3037T (intron)	SL: 10 wk, 12 wk	

***IGF1R***	10	A17299834G (5’UTR)	LL: 8 wk	9

***GHR***	Z	G6631778A (3’UTR)	HW; BW: 5 wk, 6 wk, 7 wk	10

***PIT1***	1	57 bp indel (intron)	HW; SL: 12 wk	8
		rs13687127 (intron)	SD: 11 wk	
		rs13687128 (exon)	BW: 4 wk; ADG: 0-4 wk	

***ATGL***	5	c.782G>A (exon)	BW: 6 wk, 7 wk, 8 wk	6

***IGFBP2***	7	G729T (intron)	BW: 7 wk	16
		C1032T (intron)	HW; BW: 2 wk	
		A663T (exon)	BW:1 wk, 2 wk, 3 wk, 5 wk, 6 wk, 7 wk; HW	
		G738A (exon)	BW:1 wk, 2 wk, 3 wk, 4 wk, 5 wk, 6 wk, 7 wk, 8 wk, 13 wk; HW	

***RB1***	1	g.39692 G>A	BW: 6 wk, 11 wk	24
		g.77260 A>G	BW: 5 wk, 6 wk, 7 wk, 8 wk, 9 wk, 10 wk, 11 wk, 12 wk	

***HMGA2***	1	rs15231472 (intron)	BW:6 wk	5
		rs13849381 (intron)	BW: 1 wk, 2 wk, 3 wk, 5 wk, 6 wk; HW	

a Names of SNPs are cited from original papers.

b BW: body weight; ADG: average daily gain; SL: shank length; BL: body length; LL: leg length; SD: shank diameter; HW: hatch weight; SIL: length of small intestine; BA: breast angle; BD: breast depth; SG: shank girth.
